# Self-reported mobility as a preoperative risk assessment tool in older surgical patients compared to the American College of Surgeons National Surgical Quality Improvement Program

**DOI:** 10.1186/s13741-018-0095-6

**Published:** 2018-06-19

**Authors:** Sunghye Kim, Rebecca Neiberg, W. Jack Rejeski, Anthony P. Marsh, Stephen B. Kritchevsky, Xiaoyan I. Leng, Leanne Groban

**Affiliations:** 1 Department of Internal Medicine, Section of General Internal Medicine, Wake Forest School of Medicine, Medical Center Boulevard, Winston-Salem, NC 27157 USA; 2 Sticht Center for Healthy Aging and Alzheimer’s Prevention, Wake Forest School of Medicine, Medical Center Boulevard, Winston-Salem, NC 27157 USA; 3 Division of Public Health Sciences, Department of Biostatistical Sciences, Wake Forest School of Medicine, 525 Vine, Winston-Salem, NC 27101 USA; 4 Department of Health and Exercise Science, Wake Forest University, PO Box 7868, Winston-Salem, NC 27109 USA; 5 Department of Anesthesiology, Wake Forest School of Medicine, Medical Center Boulevard, Winston-Salem, NC 27157-1009 USA

**Keywords:** Preoperative, Risk calculator, NSQIP, Mobility

## Abstract

**Background:**

The American College of Surgeons National Surgical Quality Improvement Program (NSQIP®) developed a surgical risk calculator using data from 1.4 million patients and including 1557 unique Current Procedural Terminology (CPT) codes. Although this calculator demonstrated excellent performance in predicting postoperative mortality, morbidity, and six surgical complications, it was not developed specifically for use in older surgical patients who have worse surgical outcomes and additional unique risk factors compared to younger adults. We aimed to test the ability of a simple self-reported mobility tool to predict postoperative outcomes in the older surgical population compared to the NSQIP.

**Methods:**

We used data from a prospective cohort study that enrolled 197 older surgical patients (≥ 69 years) undergoing various elective surgeries and assessed 30-day surgical outcomes. Statistical models included data from the Mobility Assessment Tool-short form (MAT-sf) alone, covariates alone, and MAT-sf data and covariates. We used leave-one-out (LOO) cross-validation of the models within our cohort and compared their performance for predicting postoperative outcomes against the NSQIP calculator based on receiver operating characteristic area under the curve (ROC AUC).

**Results:**

Patients with poor self-reported mobility experienced higher rates of postoperative complications and nursing home placement. There was no difference in performance between any of our models and the NSQIP calculator (*p* > 0.1), with AUC between 0.604 and 0.697 for predicting postoperative complications and 0.653 and 0.760 for predicting nursing home placement. All models also predicted a length of stay (LOS) similar to the actual LOS.

**Conclusion:**

Mobility assessment alone using MAT-sf can predict postoperative complications, nursing home placement, and LOS for older surgical patients, with accuracy comparable to that of the NSQIP calculator. The simplicity of this noninvasive risk assessment tool makes it an attractive alternative to the NSQIP calculator that requires 20 patient predictors and the planned procedure, or CPT code to predict the chance that patients will have 15 different adverse outcomes following surgery.

## Background

The American College of Surgeons National Surgical Quality Improvement Program (NSQIP®) surgical risk calculator (American College of Physicians n.d.), released in 2013 (Bilimoria et al. 2013), was developed using data from more than 1.4 million patients, encompassing 1557 unique Current Procedural Terminology (CPT) codes. Patient-related preoperative variables include age, sex, functional status, American Society of Anesthesiologists (ASA) classification, steroid use for chronic conditions, ascites within 30 days prior to surgery, systemic sepsis within 48 h prior to surgery, ventilator dependency, disseminated cancer, diabetes (DM), hypertension (HTN), congestive heart failure 30 days prior to surgery, dyspnea, current smoker within 1 year, history of severe chronic obstructive pulmonary disease (COPD), dialysis, acute renal failure, and body mass index (BMI). The calculator also takes into account the type of surgery, based on specific CPT codes and emergency status (American College of Physicians n.d.). Although the NSQIP surgical risk calculator demonstrated excellent performance in predicting postoperative mortality (c-statistic = 0.944), morbidity (c-statistic = 0.816), and six surgical complications (c-statistics > 0.8), it was not developed specifically for use in the older patient population, which is rapidly growing in the USA and is known to have worse surgical outcomes than younger patients (Sukharamwala et al. 2012; Raats et al. 2015; Bentrem et al. 2009). Specifically, the NSQIP surgical risk calculator does not include factors such as frailty and mobility that are known to be important predictors of surgical outcomes in older patients (Kim et al. 2016; Makary et al. 2010).

The NSQIP and the American Geriatric Society published a best practice guideline for optimal preoperative assessment of older surgical patients (Chow et al. 2012). The guideline recommends assessment of patients’ gait and mobility impairment and fall risk, using tests such as the Timed Up and Go test to assess surgical risk in older patients. While the NSQIP surgical risk calculator does include patients’ functional status as a categorical variable with three options (independent, partially dependent, and totally dependent), this variable does not capture the various degrees of functional limitation that older patients experience. Furthermore, the NSQIP surgical risk calculator does not take into account patients’ mobility status.

In a prospective study of older surgical patients, we assessed preoperative self-reported mobility using a novel tool, the Mobility Assessment Tool-short form (MAT-sf), and found it to be a good predictor for postoperative complications, hospital length of stay (LOS), and nursing home placement (Kim et al. 2016). Here, we used data from that study to develop a simplified preoperative risk assessment model that includes measures of self-reported mobility to predict postoperative outcomes in older patients. The aim of this study was to compare the performance of these simple surgical risk calculators to the NSQIP surgical risk calculator in predicting postoperative outcomes among older patients.

## Methods

### Study design

We previously conducted a prospective cohort study of older patients (≥ 69 years) who were undergoing elective, noncardiac surgery from July 2012 to February 2014 (Kim et al. 2016). Eligible patients were asked to provide written informed consent (IRB approval number 000193921; 4/23/2012) before undergoing standardized assessments. Preoperative risk factors, including comorbidity, BMI, and ASA Physical Status Classification, were obtained at enrollment. Self-reported mobility was assessed using the MAT-sf, administered by trained study personnel. The MAT-sf is a 10-item, computer-based assessment of mobility using animated video clips (Rejeski et al. 2013). The 10 items ask patients to report on their ability to perform a broad range of functions, including walking on level ground, slow jogging, walking outdoors on uneven terrain, walking up a ramp with and without use of a handrail, stepping over hurdles, ascending and descending stairs with and without use of a handrail, and climbing stairs while carrying bags (Rejeski et al. 2010a). Each item is accompanied by an animated video clip together with the responses for that question (number of minutes, number of times, yes/no). The MAT-sf was validated by two separate stepwise regression analyses, one for the Short Physical Performance Battery (SPPB) and a second for the 400-m walk. In these analyses, the Pepper Assessment Tool for Disability (PAT-D) mobility score was entered first followed by the MAT-sf scores. In both analyses, the entry of the MAT-sf contributed over and above the PAT-D mobility subscale to the explanation of performance-based function; for the SPPB, the change in *R*^2^ was an additional 9.8% and for the 400-m walk it was 16.7%. The zero order correlations of the MAT-sf to the SPPB and 400-m walk gait speed were 0.59 (*p* < 0.001) and 0.58 (*p* < 0.001), respectively. It is also of interest to point out the standardized *β* weight for the MAT-sf was substantially larger than the PAT-D mobility subscale in both analyses (Rejeski et al. 2013; Rejeski et al. 2010b).

Postoperative outcomes were assessed by medical record review, including postoperative complications within 30 days of the operation, LOS, and nursing home placement. The NSQIP definition of postoperative complications was used and included surgical site infection (superficial, deep, and organ space), wound disruption, pneumonia, unplanned intubation, thromboembolism, on ventilator > 48 h, urinary tract infection, progressive renal insufficiency, acute renal failure, cerebrovascular accident, cardiac arrest, myocardial infarction, and sepsis (Khuri et al. 1998).

### Statistical analysis

Sex differences in MAT-sf scores were compared using Student’s *t* test. Percentages of postoperative complications and nursing home placement among patients by sex-specific mobility tertile (best, mid, and worst tertiles of MAT-sf score) were compared using chi-square tests. Length of stay was compared across MAT-sf tertiles using the Wilcoxon rank sum test.

To compare our risk models to the ASC NSQIP surgical risk calculator, we first calculated predicted surgical outcomes using risk factors obtained at enrollment by the NSQIP calculator (American College of Physicians n.d.). Next, logistic regression models were used to estimate probabilities of postoperative complications and nursing home placement and a Poisson regression model was used to estimate LOS for each participant. Three sets of models were created including (1) MAT-sf scores alone; (2) covariates alone: age, sex, BMI, ASA status, DM, HTN, and surgical risk; and (3) both the covariates and MAT-sf scores. Since the NSQIP surgical risk calculator estimates of LOS and probability of postoperative complications and nursing home placement are predictions, and we already knew these outcomes for our cohort, we utilized the leave-one-out (LOO) cross-validation method when fitting our models to obtain model-based predictions for the observation left out. Finally, the receiver operating characteristic (ROC) areas under the curve (AUCs) were calculated for estimated probabilities of postoperative complications and nursing home placement from both the NSQIP surgical risk calculator and our models. Estimates of AUC standard errors and confidence intervals for all models and the NSQIP surgical risk calculator were obtained, and comparisons of AUCs were done through a nonparametric approach using the SAS ROC macro (SAS Institute, Cary, NC) (DeLong et al. 1988). For LOS, the estimates between each of the models and the actual LOS were compared in a mixed model ANOVA accounting for correlation between measurements on the same patient. The Spearman’s rank correlation was also used to test associations between the MAT-sf and the NSQIP-calculated length of stay, any complications, and probability of nursing home placement.

## Results

### Patient cohort

For this study, we utilized data from a total of 197 patients undergoing elective, noncardiac surgery who were enrolled in a prospective trial (Kim et al. 2016). The mean (± SD) age was 75.2 years (± 5.0), 51% of the patients were female, and the cohort was predominantly white (Table 1). After surgery, 30 (15.2%) of the patients had postoperative complications within 30 days of the operation, while 27 (13.7%) were placed to nursing homes. The median (interquartile range [IQR]) LOS was 3.0 (2.0–4.0) days and the mean ± SD was 3.6 ± 4.2 days.

**Table 1:** Characteristics of patient cohort (*n* = 197)

Age, mean (SD)	75.2 (5.0)
Female, *n* (%)	101 (51)
Race, *n* (%)	
White	179 (91)
African American	15 (8)
Other	3 (1.5)
Body mass index, kg/m^2^, mean (SD)	27.8 (5.6)
ASA physical status, *n* (%)	
I	0 (0)
II	47 (24)
III	136 (69)
IV	14 (7)
Surgical risk, *n* (%)	
Low	35 (18)
Intermediate-to-high	162 (82)
Mobility Assessment Tool-short form score, median (IQR)	53.1 (46.4–61.6)

*ASA* American Society of Anesthesiologists, *IQR* interquartile range, *SD* standard deviation

### Mobility measured by MAT-sf

**Fig. 1:**
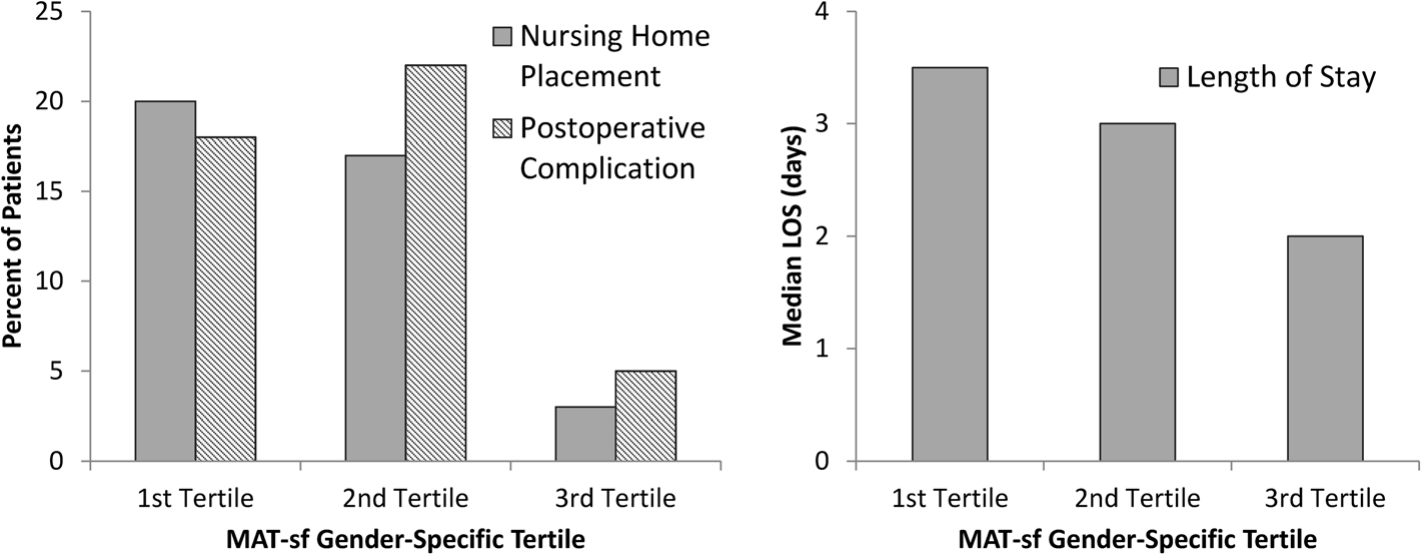
Rates of postoperative complications, nursing home placement, and hospital length of stay per gender-specific Mobility Assessment Tool-short form tertile, (*p* = 0.014, *p* = 0.009, *p* < 0.0001, respectively)

As we have reported previously, the median MAT-sf score was 53.1 (IQR 46.4–61.6). Men had higher MAT-sf scores (58.3 [IQR 48.3–65.5]) than women (49.9 [IQR 42.2–55.5]), *p* < 0.001. Patients in lower sex-specific MAT-sf tertiles had higher rates of postoperative complications (*p* = 0.014) and nursing home placement (*p* = 0.009), as well as longer LOS (*p* < 0.0001; Fig. 1).

### Comparison of new models and NSQIP surgical risk score

**Fig. 2:**
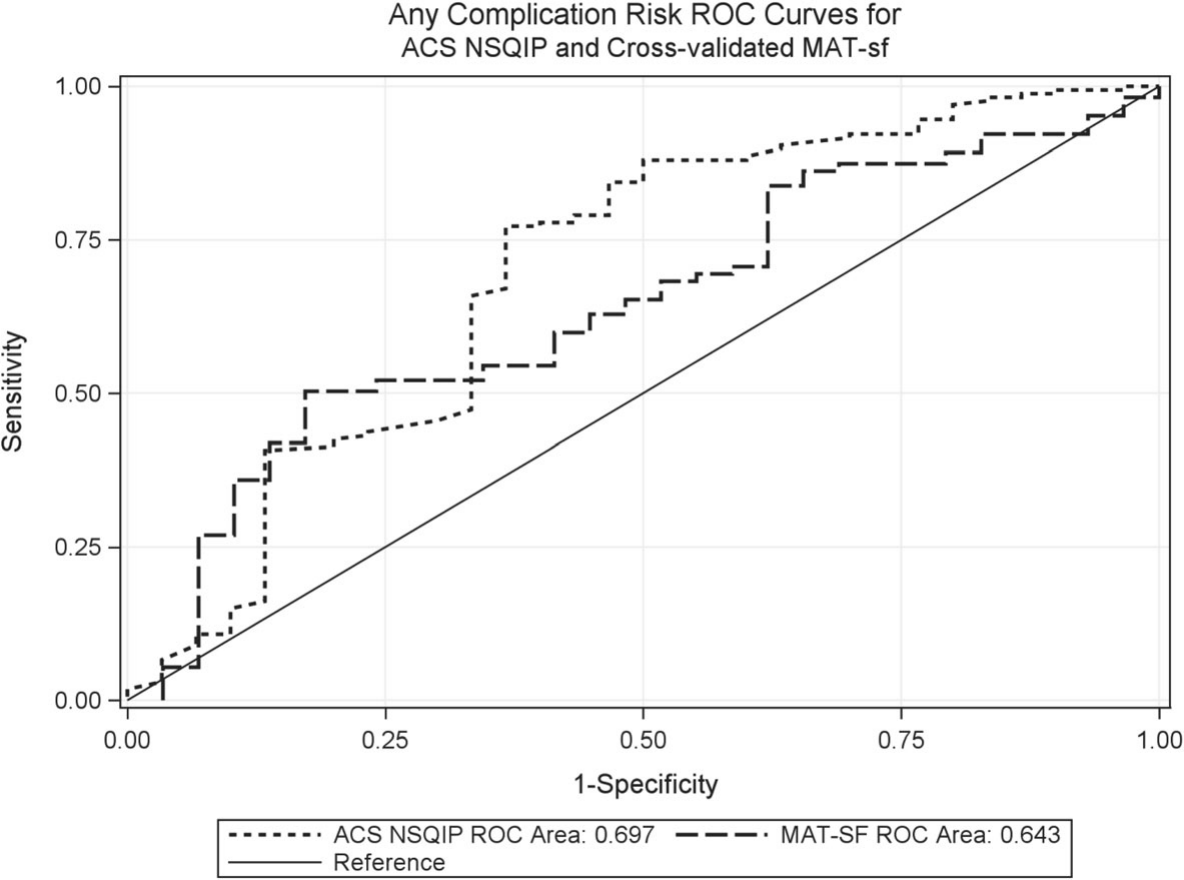
Area under the curve for predicting postoperative complications using the American College of Surgeons National Surgical Quality Improvement Program surgical risk score and cross-validated Mobility Assessment Tool-short form only model, *p* = 0.54

**Fig. 3:**
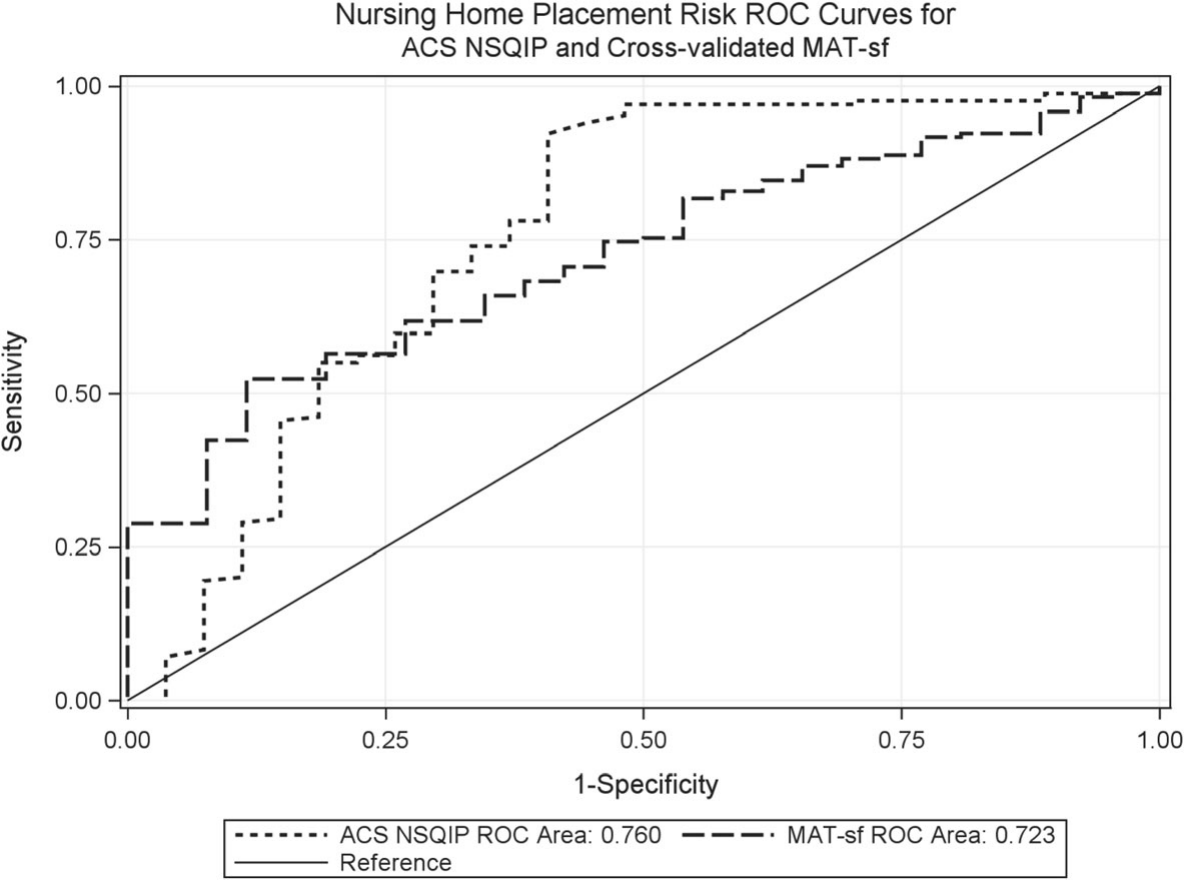
Area under the curve for predicting nursing home placement using the American College of Surgeons National Surgical Quality Improvement Program surgical risk score and cross-validated Mobility Assessment Tool-short form only model, *p* = 0.58

We developed and cross-validated models using MAT-sf only, covariates only (age, gender, BMI, ASA status, DM, HTN, and surgical risk), and MAT-sf plus covariates to predict postoperative outcomes. Table 2 summarizes the ROC AUC of each cross-validated model and the NSQIP surgical risk score for the outcomes of postoperative complications and nursing home placement. There was no significant difference in AUC between any of the new models and the NSQIP surgical risk score for either postoperative complications or nursing home placement. Figure 2 illustrates the AUC of the MAT-sf only model and the NSQIP surgical risk score for postoperative complications (AUC 0.643 [95% CI 0.538–0.748] and 0.697 [0.580–0.813], respectively, *p* = 0.54). Figure 3 illustrates the AUC of the MAT-sf only model and the NSQIP surgical risk score for nursing home placement (AUC 0.723 [0.632–0.813] and 0.760 [0.640–0.880], respectively, *p* = 0.58). Table 3 lists the estimated LOS based on our three models and the NSQIP surgical risk score. There were no significant differences between the actual LOS in the cohort and LOS estimated using our three models or the NSQIP surgical risk score. The correlations between the MAT-sf and NSQIP-calculated length of stay, any complications, and probability of nursing home placement are shown in Table 4. There was a significant association between these two scoring systems for postoperative outcomes.

**Table 2:** Comparisons of cross-validated models and NSQIP® surgical risk score for predicting postoperative outcomes in older patients

Outcomes	Model	ROC AUC (95% CI)	Estimate of difference (95% CI)*	*p* value*
Postoperative complications	MAT-sf only	0.643 (0.538–0.748)	− 0.054 (− 0.227, 0.120)	0.54
	Covariates only†	0.604 (0.496–0.711)	− 0.093 (− 0.232, 0.046)	0.19
	MAT-sf + covariates†	0.641 (0.529–0.753)	− 0.056 (− 0.203, 0.092)	0.46
	ACS NSQIP surgical risk score	0.697 (0.580–0.813)		
Nursing home placement	MAT-sf only	0.723 (0.632–0.813)	− 0.037 (− 0.167,0.094)	0.58
	Covariates only†	0.653 (0.543–0.762)	− 0.107 (− 0.238,0.024)	0.11
	MAT-sf + covariates†	0.708 (0.596–0.821)	− 0.051 (− 0.184, 0.081)	0.45
	NSQIP surgical risk score	0.760 (0.640–0.880)		

*NSQIP®* National Surgical Quality Improvement Program, *AUC* area under the curve, *MAT-sf* Mobility Assessment Test-short form, *ROC* receiver operator characteristic

*Comparing each model to NSQIP surgical risk score

†Covariates include age, gender, body mass index, ASA status, diabetes mellitus, hypertension, and surgical risk

**Table 3:** Comparison of actual and model-based estimates of postoperative hospital length of stay in older patients

Model	Predicted LOS (mean ± SD)	Difference from actual LOS (95% CI)	*p* value*
Mobility Assessment Tool-short form only	3.55 ± 0.76	− 0.04 (− 0.61–0.54)	0.90
Covariates only†	3.59 ± 1.54	− 0.00 (− 0.41–0.42)	0.99
Mobility Assessment Tool-short form + covariates†	3.55 ± 1.59	− 0.03(− 0.59–0.53)	0.92
NSQIP surgical risk score	3.36 ± 2.40	− 0.22(− 0.78–0.33)	0.43
Actual LOS	3.58 ± 4.15	–	

*LOS* length of stay, *NSQIP®* National Surgical Quality Improvement Program, *SD* standard deviation

*Comparing estimated length of stay from each model and NSQIP surgical risk score to actual length of stay

†Covariates include age, gender, body mass index, ASA status, diabetes mellitus, hypertension, and surgical risk

**Table 4:** Spearman’s correlation between MAT-sf and NSQIP calculator predicted outcomes

Spearman correlation coefficients Prob > |*r*| under H0: Rho = 0				
	MAT-sf	LOS	Any complications	Nursing home placement
MAT-sf	1.00000	− 0.33034 < 0.0001	− 0.16252 0.0229	− 0.44940 < 0.0001
Length of stay	− 0.33034 < 0.0001	1.00000	0.69552 < 0.0001	0.67603 < 0.0001
Any complications	− 0.16252 0.0229	0.69552 < 0.0001	1.00000	0.25218 0.0004
Nursing home placement	− 0.44940 < 0.0001	0.67603 < 0.0001	0.25218 0.0004	1.00000

*MAT-sf* Mobility Assessment Test-short form, *LOS* length of stay

## Discussion

We set out to develop a surgical risk calculator that was simple and specific to the older surgical patient population. We developed three models using data from our prospective cohort of 197 older surgical patients and cross-validated the models to provide robust probabilities of risk. The model using only the MAT-sf score had similar ability for predicting postoperative complications and nursing home placement as the NSQIP surgical risk calculator. Thus, the MAT-sf score provides a simple, easy to use tool to predict surgical outcomes in older patients. The model performed as well as the NSQIP surgical risk calculator in predicting LOS.

The NSQIP risk calculator was developed using data from more than 1.4 million patients and has demonstrated excellent performance in predicting postoperative outcomes (American College of Physicians n.d.; Bilimoria et al. 2013). However, the NSQIP calculator was not specifically developed for use in an older patient population, in which unique factors, such as mobility, are important predictors of surgical outcomes. There have been efforts to develop simpler tools to predict postoperative outcomes in the older patient population. A few studies have used mobility as a marker of postoperative outcomes. Robinson et al. tested the Timed Up and Go test as a predictive tool among 272 older patients (98 patients undergoing colorectal surgery and 174 patients undergoing cardiac surgery). The Timed Up and Go test measures the time it takes for an individual to rise from a chair, ambulate 10 ft, turn around a cone marker, return to the chair, and sit back down. In patients undergoing either colorectal or cardiac surgery, the study reported the ROC AUC for predicting postoperative complications using the Timed Up and Go test as 0.775 and 0.684, respectively, compared to 0.554 and 0.552 for standard-of-care surgical risk calculators (Robinson et al. 2013). The MAT-sf tool had similar ROC AUCs for postoperative complications as those reported for the Timed Up and Go test, and we did not see any difference between the MAT-sf tool and the NSQIP surgical risk calculator. One important distinction between these two simple perioperative risk score measures is that the Timed Up and Go is a performance test of physical function whereas the MAT-sf is a self-report measure. Consequently, the Timed Up and Go might not be as feasible due to space and patient limitations. Since the study by Robinson et al. limited the enrollment criteria to patients who were undergoing colorectal or cardiac surgery and analyzed them separately, the risk associated with each type of surgery could be controlled. By contrast, our study enrolled any older patients who were undergoing elective surgery (Table 5). Given the relatively small sample size of our study, we could not control for the risk associated with each type of surgery. In the models that used the covariates alone or covariates plus MAT-sf, we controlled for the surgical risk in terms of low vs. intermediate-to-high risk using the definitions from American College of Cardiology/American Heart Association guidelines (Fleisher et al. 2014).

**Table 5:** Summary of surgical procedures by sex

Surgery	Male	Female	Total
Orthopedic surgery	42	72	114
Hip	13	26	
Knee	9	18	
Spine	18	21	
Other	2	7	
Urology	25	4	29
Kidney	5	2	
Prostate	10	0	
Other	10	2	
Intraperitoneal	13	7	20
Colorectal	5	4	
Hernia	3	1	
Other	5	2	
Otolaryngology	5	8	13
Thyroid	0	3	
Other	5	5	
Vascular	6	3	9
Carotid endarterectomy	4	1	
Other	2	2	
Gynecology	0	4	4
Hysterectomy	0	2	
Other	0	2	
Neurosurgery	2	1	3
Pituitary	1	1	
Other	1	0	
Other	3	2	5

Reddy and colleagues examined the ability of a stair climbing activity to predict surgical outcomes in 264 patients ≥ 19 years of age who were undergoing elective abdominal surgery. They reported that stair climbing predicted morbidity better than the NSQIP surgical risk calculator (AUC = 0.81 vs. 0.62, *p* < 0.0001). However, the stair climbing task caused physiologic stress, especially in those patients who were slower climbers, causing a 16.6 and 21.8% increase in heart rate and mean arterial pressure, respectively, compared to 7.4 and 7.9% for faster climbers (Reddy et al. 2016). Although this study demonstrated excellent power of stair climbing time as a tool to predict postoperative outcomes, it is an even more intensive test than the Timed Up and Go test, with higher risk (including falls and cardiac events with physiologic stress). Also, the time and space constraints, as well as personnel requirements, clearly limit the feasibility of the stair climbing test in clinical practice.

What is noteworthy is that our models were not different from the NSQIP surgical risk calculator in predicting postoperative hospital LOS. Indeed, the biggest factor that determines postoperative hospital LOS is the type of surgery; for example, patients who undergo laparoscopic cholecystectomy will have a much shorter LOS than patients who undergo open hemicolectomy. The NSQIP surgical risk calculator was developed using 1557 unique CPT codes and allows input of a single procedure CPT code. Even though our models did not include the surgical risk in the model (e.g., MAT-sf only model) or reflect the risks fully (only two degrees of surgical risks [low vs. intermediate-to-high]), the predictions for LOS using the simple MAT-sf model were remarkably similar to the universal calculator. Adequately incorporating procedure complexity and risk into future studies that use the simple MAT-sf could solidify its efficacy in forecasting surgical outcomes.

The limitations of the study include its single-center design and use of a majority white patient cohort, features of the study design that compromise the external validity of the findings. We were unable to include the full list of measures included in the NSQIP calculator due to our small sample size and lack of variability in some measures such as cancer and chronic heart failure, among others. Since this issue could have accounted for the similarities observed between the universal calculator and the MAT-sf models, the results must be interpreted with caution. Nonetheless, we expect that our estimation of postoperative risks and LOS with the MAT-sf tool may have been better if we had a larger sample with increased variability. Although we used the LOO cross-validation method, our findings still need to be tested in a separate cohort. Lastly, the models were developed from relatively small number of older patients (*n* = 197), which may not accurately reflect surgical risk.

## Conclusion

The MAT-sf can predict postoperative outcomes (complications, LOS, and nursing home placement) in older surgical patients with predictive performance comparable to the NSQIP surgical risk calculator. Given the simplicity and noninvasive nature of the MAT-sf tool, it can be easily adopted into clinical practice for preoperative risk assessment. Future studies in a larger cohort of patients undergoing a single type of surgery are warranted to validate this new method to predict postoperative outcomes.

## Abbreviations

ASA: American Society of Anesthesiologists
AUC: Area under the curve
BMI: Body mass index
COPD: Chronic obstructive pulmonary disease
CPT: Current Procedural Terminology
DM: Diabetes mellitus
HTN: Hypertension
IQR: Interquartile range
LOO: Leave one out
LOS: Length of stay
MAT-sf: Mobility Assessment Tool-short form
NSQIP®: National Surgical Quality Improvement Program
ROC: Receiver operating characteristic
